# COVID-19 and neurological disorders: what might connect Parkinson’s disease to SARS-CoV-2 infection

**DOI:** 10.3389/fneur.2023.1172416

**Published:** 2023-05-18

**Authors:** Salvatore Iacono, Giuseppe Schirò, Chiara Davì, Sergio Mastrilli, Michelle Abbott, Fabrizio Guajana, Valentina Arnao, Paolo Aridon, Paolo Ragonese, Cesare Gagliardo, Claudia Colomba, Nicola Scichilone, Marco D’Amelio

**Affiliations:** ^1^Department of Biomedicine, Neuroscience, and Advanced Diagnostics, University of Palermo, Palermo, Italy; ^2^Azienda Ospedaliera Universitaria Policlinico Paolo Giaccone di Palermo, Palermo, Italy; ^3^Department of Health Promotion, Mother and Child Care, Internal Medicine and Medical Specialties, University of Palermo, Palermo, Italy; ^4^UO Neurologia e Stroke Unit, Azienda di Rilievo Nazionale ad Alta Specializzazione, Ospedali Civico Di Cristina Benfratelli, Palermo, Italy; ^5^Division of Respiratory Diseases, Department of Health Promotion Sciences, Maternal and Infant Care, Internal Medicine and Medical Specialties (PROMISE), University of Palermo, Palermo, Italy

**Keywords:** Parkinson’s disease, COVID-19, SARS-CoV-2, risk factors, neurodegenerative diseases, neuroepidemiology, dual hit hypothesis, social restriction

## Abstract

SARS-CoV-2 infection leading to Coronavirus disease 19 (COVID-19) rapidly became a worldwide health emergency due to its elevated infecting capacity, morbidity, and mortality. Parkinson’s disease (PD) is the second most common neurodegenerative disorder and, nowadays the relationship between SARS-CoV-2 outbreak and PD reached a great interest. Apparently independent one from the other, both diseases share some pathogenetic and clinical features. The relationship between SARS-CoV-2 infection and PD is complex and it depends on the direction of the association that is which of the two diseases comes first. Some evidence suggests that SARS-CoV-2 infection might be a possible risk factor for PD wherein the exposure to SARS-CoV-2 increase the risk for PD. This perspective comes out from the increasing cases of parkinsonism following COVID-19 and also from the anatomical structures affected in both COVID-19 and early PD such as olfactory bulb and gastrointestinal tract resulting in the same symptoms such as hyposmia and constipation. Furthermore, there are many reported cases of patients who developed hypokinetic extrapyramidal syndrome following SARS-CoV-2 infection although these would resemble a post-encephalitic conditions and there are to date relevant data to support the hypothesis that SARS-CoV-2 infection is a risk factor for the development of PD. Future large, longitudinal and population-based studies are needed to better assess whether the risk of developing PD after COVID-19 exists given the short time span from the starting of pandemic. Indeed, this brief time-window does not allow the precise estimation of the incidence and prevalence of PD after pandemic when compared with pre-pandemic era. If the association between SARS-CoV-2 infection and PD pathogenesis is actually putative, on the other hand, vulnerable PD patients may have a greater risk to develop COVID-19 being also more prone to develop a more aggressive disease course. Furthermore, PD patients with PD showed a worsening of motor and non-motor symptoms during COVID-19 outbreak due to both infection and social restriction. As well, the worries related to the risk of being infected should not be neglected. Here we summarize the current knowledge emerging about the epidemiological, pathogenetic and clinical relationship between SARS-CoV-2 infection and PD.

## Parkinson’s disease and infectious diseases

Parkinson’s disease (PD) is the most common worldwide neurodegenerative disorder after Alzheimer’s disease, characterized by an early and dramatic loss of dopaminergic neurons in substantia nigra, leading to motor and non-motor symptoms (1). Up to date, the pathogenesis of PD is not fully understood, and it is thought to be based on a subtle interplay between genetic and environmental risk factors (2). Age represents the greatest risk factor for the development of PD and because of the aging of the population as well as the longer life expectancy, the prevalence of PD is rapidly increasing; it has been estimated that the number of people with PD will double to 50% in 2030 until a prevalence between 8.7 and 9.3 millions of affected individuals (3). Although demographic and genetic factors in the pathogenesis of PD are well characterized, the role of environmental exposures and particularly that of infectious disease are not clear. Many factors have been associated with PD, some are directly associated with an increased risk (e.g., pesticide, methamphetamine, melanoma, heavy metal exposure) (4), while others seem to be somehow protective (e.g., alcohol consumption, caffeine, estrogen) that is inversely associated with its risk (5–8). Among others, infections have been suggested as possible risk factor for idiopathic PD and secondary parkinsonism. This suggestion originates both from the description of the post-encephalitic parkinsonism occurred after the influenza pandemic in 1918, and from the observation of the parkinsonian cluster’s phenomenon (9). According to this hypothesis, the risk of developing PD is greater in people who share close quarters such as doctors, teachers, nurses thus indicating a common and environmental risk factor such as viral infections (10). In fact, some researchers suggest a direct contribution of the viral infection or of the establishment of post-infectious mechanisms following a viral infection in neurodegeneration (11). In this sense, people in contact with the public, the reservoir of the infection, could be at greater risk. Furthermore, the role of the infections in the pathogenesis of PD might be supported by the Braak hypothesis identifying olfactory bulbs and peripheral nerves of the gastrointestinal tract as starting points of the disease and portal entry for toxins and infectious agents (12). Recent meta-analysis indicated that infection was associated with an increased risk of developing PD by 20% compared to controls with a marked effect for bacterial infection (Odds ratio [OR] = 1.4) and less for viral infections (OR = 1.09) (13). However, a distinction should be made between primary PD (i.e., idiopathic) and secondary parkinsonism (i.e., post-infective) due to infectious disease. Indeed, it has been reported that influenza virus infection is the most common viral infection associated with risk of developing parkinsonism, while hepatitis C seems to be associated with an increased risk to develop PD, but no risk was found for hepatitis B (14). Also, though parkinsonism is well documented in patients with chronic human immunodeficiency (HIV) virus infection and HIV-associated neurocognitive disorders, several evidence did not support HIV as a risk factor for the development of PD (15). Some acute viral infection may directly involve the basal ganglia such as Japanese Encephalitis virus, Coxsackie virus, Western equine Encephalitic virus and West Nile virus leading to parkinsonism but their association with PD has never been established (9). Recently, a meta-analysis has shown that the development of parkinsonism may be an underdiagnosed complication of Dengue virus infection. However, also in this case, as for the other viruses mentioned above, there seems to be no data showing an association between an increased risk of developing idiopathic PD and a previous infection by Dengue virus (16). Antigenic mimicry has been hypothesized to explain the increased risk of PD after herpes simplex virus 1 (HSV-1) infection. Indeed, antibodies to HSV-1 are able to cross-react against some epitopes of the α-synuclein, suggesting the possibility of promoting the aggregation of α-synuclein (17). The persistence of movement disorders about 3-5 years after acute infection by Japanese encephalitis B has been associated with lesions of substantia nigra detected by neuroimaging studies (11).

**Figure 1:**
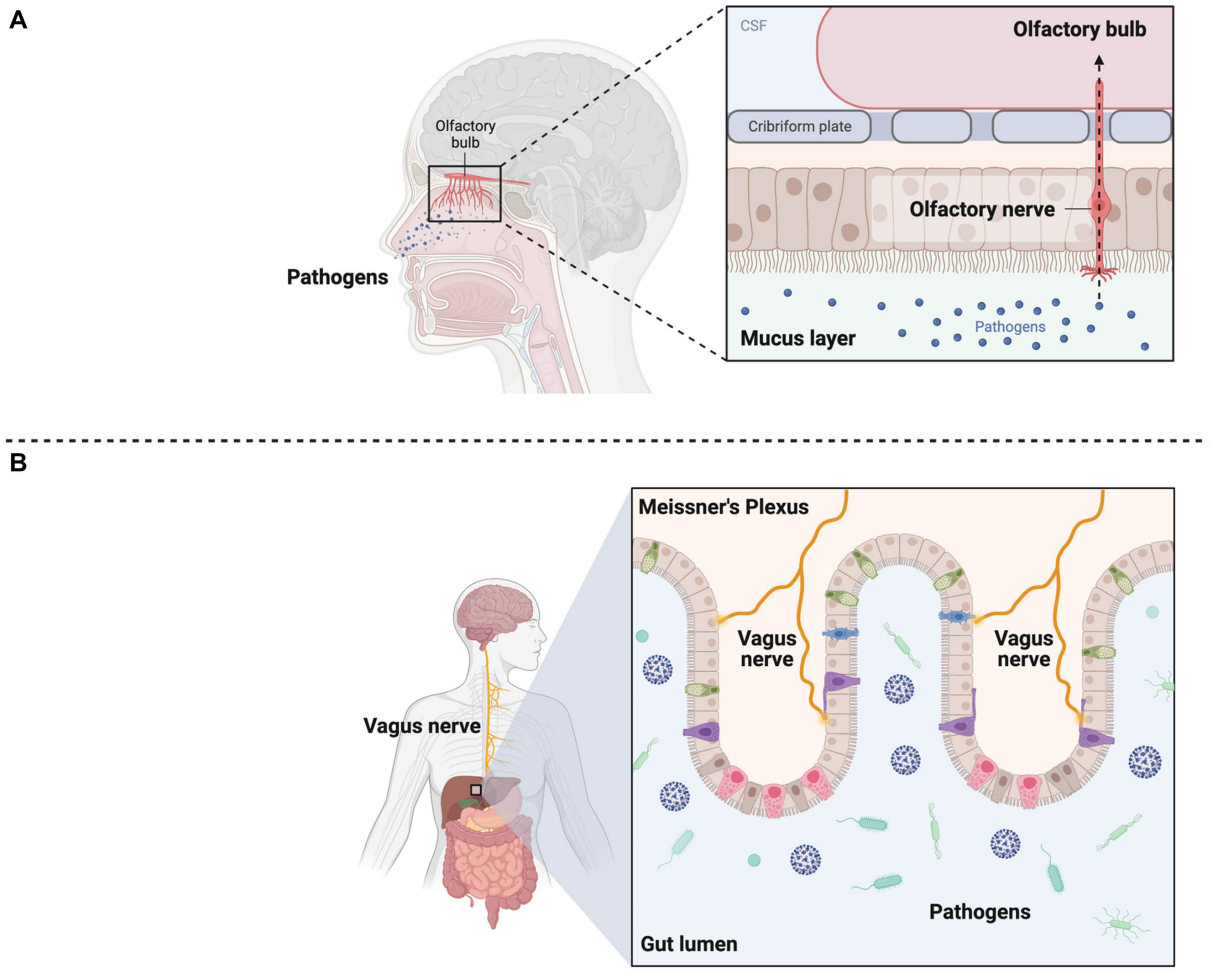
Graphical representation of dual hit hypothesis. **(A)** Pathogens enter the brain through olfactory epithelium being transported via anterograde transport throughout olfactory nerve. **(B)** pathogens in the intestinal lumen go toward vagus nerve terminations in the Meissner’s plexus being transported via retrograde transport throughout vagus nerve until brainstem. The term dual hit comes out from the two-way pathogenetic access of pathogen to the brain. Created with BioRender.com.

Among bacteria, a recent meta-analysis showed as *H. pylori* increase the risk by 1.5-2-fold to develop PD (18). Other study showed that seropositive patients for at least five or six among Cytomegalovirus, Epstein–Barr virus, Herpes simplex-1, *Borrelia burgdorferi*, *Chlamydia pneumoniae* and *H. pylori* are at more risk to develop PD compared to healthy controls (19). Despite the high amount of literature published, the relationship between infectious diseases and the pathogenesis of PD is still controversial. This point needs to be clarified in order to prevent and treat the potentially curable factors causing PD. The relevance of the association between infectious disease and PD reaches great interest in the light of the Severe Acute Respiratory Syndrome Coronavirus 2 (SARS-CoV-2) outbreak, leading to Coronavirus Disease 2019 (COVID-19). Despite COVID-19 usually presents with fever, cough and dyspnea, a wide range of neurological manifestations are currently reported (20–22). Of note, Rao et al. recently reported three cases of parkinsonism onset after SARS-CoV-2 infection characterized by orthostatic hypotension, bradykinesia and rigidity few days after symptomatic COVID-19 although these were recognized as post-encephalitic parkinsonism that is far from PD diagnosis (23). Thus, a clear distinction should be served between post-infective parkinsonism and degenerative PD as a consequence of SARS-CoV-2 infection. If the former is well-described in literature, latter is actually hypothetical, and it is almost based on the dual hit hypothesis (12). In this view, it has been hypothesized that neurotrophic pathogen (e.g., viral) enters the brain via the olfactory bulb or Meissner’s plexus being transported anterogradely or retrogradely into temporal lobe and brainstem, through olfactory and vagus nerves, respectively (Figure 1). When the pathogen reaches the midbrain until substantia nigra, the typical aspect of PD will be unmasked. The term “dual hit” comes out from the two-way pathogenetic access of pathogen to the brains (i.e., nasal and gastrointestinal) and also it may explain the prodromal and non-motor symptoms of PD such as hyposmia and autonomic dysfunction (12).

However, it is not negligible the detrimental impact of pandemic on the clinical course and therapeutical management of PD patients. In this review we will summarize the current literature in the field of pathogenetic, clinical and therapeutic points of view about the relationship between COVID-19 and PD.

## Coronavirus and nervous system: historical notes

The potential role of the Coronaviruses to determine nervous system diseases in humans has been recognized nearly 40 years ago, when two coronaviruses were isolated from brain material obtained from the autopsy of two patients affected by multiple sclerosis (24). Successively, a possible role for Coronaviruses in the pathogenesis of postencephalitic parkinsonism has been postulated when mice infected with mouse hepatitis virus (i.e., MHV-A59) showed a strong tropism for the basal ganglia (25). Few years later, a study on samples of cerebrospinal fluid obtained from the tissue bank at the Neurological Institute of Columbia Presbyterian Hospital reported, in patients with PD compared to normal age-matched controls, an elevated CSF antibody response to two Coronaviruses (i.e., MHV-JHM and MHV-A59) (26). MHV-A59 infected C57BL/6 mice showed a viral presence in the brain localized mainly in the subthalamic substance and in the subthalamic nucleus with neuronal loss, gliosis and cellular vacuolization. Furthermore, the MHV-A59 genome appears to persist for many months in the CNS after infection (25).

Looking at more recent years, it was 2002 when an epidemic of a Coronavirus starting in Asia and spreading to throughout the world, characterized by severe acute respiratory syndrome was complicated by a range of neurological disorders interesting either CNS or PNS (27, 28). Similarly, ten years later, a different Coronavirus namely Middle East Respiratory Virus (MERS) spread in the Middle East. Although intranasal administration of the virus in experimental models is followed by subsequent infection of the brain, the presence of the virus has never been demonstrated in the CSN of human patients (29, 30). SARS-CoV-2 is characterized by a high homology both with coronavirus of first epidemic as well as with MERS, and it seems to be able to cause injury both to the central and the peripheral nervous systems. It should be noted that the Coronaviruses possess not only neurotropism but also important neurotoxic properties. In fact, starting with the first reported case of acute disseminated encephalomyelitis (ADEM) (31), there are other several reports that have observed extensive demyelination of the CNS following infection by coronaviruses. For instance, Sars-CoV-2 infection has shown to be associated with ADEM in patients with COVID-19 (32). The occurrence of encephalitis during Coronaviruses infection is also reported, for example, in murine models infected with HCoV-OC43 (33) or in patients with COVID-19 in which is described a broad spectrum of encephalitic manifestations (34).

## Prevalence and outcome of COVID-19 in PD patients

Patients with PD are vulnerable, and they may have a higher risk to contract SARS-CoV-2 infection as well as they may expedience a more severe course of the infection. On the other hand, the shared clinical and pathogenetic features between SARS-CoV-2 infection and PD suggests that infection may increase the risk of developing PD. In this view, the studies exploring the incidence and prevalence as well as the magnitude of COVID-19 in patients with PD are relevant. In the early period of pandemic era, Antonini et al. revealed that a longer PD duration was associated with poorer outcomes from COVID-19 with high rate of mortality (35). Another earlier study showed as mild-to-moderate COVID-19 was contracted independently of age and disease duration in PD patients as well as the outcome from the infection was similarly between mild-stage PD and healthy population (36). Some isolated case reports of patients who developed a Parkinsonian syndrome with neuroimaging evidence of nigrostriatal dopaminergic system deficiency following SARS-CovV2 infection and development of COVID-19 have been reported (11, 37). Though numerous evidence would support the hypothesis that COVID-19 are associated with an increased risk of PD, a recent study showed that number of patients with parkinsonism diagnosed within 6 months after COVID-19 was low (0.46%) (38). The uncertainty about the pre-infection neurological status of patients who experienced parkinsonism or PD after COVID-19 is a crucial issue regarding the possibility to attribute a causality relationship between COVID-19 and PD. Indeed, COVID-19 may only unmask an underlying preclinical PD (39). On the other hand, some authors hypothesizes that PD may serve as protective factors against SARS-CoV-2 infection since the neurodegenerative process occurring in PD may disrupt the gateway of the virus and its retrograde diffusion toward olfactory and vagus nerves (40). A recent survey showed that the prevalence of COVID-19 was similar comparing PD patients and healthy subjects (7.1 vs. 7.6%, respectively). However, in this study the clinical expression of COVID-19 in patients with PD was similar to non-PD patients though the former were less likely to report shortness of breath and required hospitalization (41). Another study reported a higher prevalence of COVID-19 in the PD population compared to that observed in the general population without a different mortality rate between PD and non-PD patients (42). More recently, a study conducted among 1,294 resident in nursing homes showed a higher risk of 30-day mortality in patient with PD compared to controls after adjustment for gender, age, and comorbidities (43). A recent metanalysis including 13 studies highlighted among PD patients affected by COVID-19, respectively, a hospitalization rate and ICU admission of 39.9 and 4.7% and a mortality rate of about 25%; these data were comparable to patients without PD (44). In USA, two studies failed to prove PD as independent risk factor for severe COVID-19 and death although the advanced disease and elderly were proposed as the main risk factors for developing severe COVID-19 (45, 46). Finally, a recent cohort study over a period of 15 months by Zenesini et al. founded a higher risk of SARS-CoV-2 infection in PD patients compared to healthy controls as well as a slightly higher risk for hospitalization with a 30-day mortality risk higher in parkinsonism (58%) than in PD (19%) and controls (26%) (47). Given that PD patients are usually older than 60 years, and that increased age is associated with death in patients with COVID-19, age and age-related comorbidity should be considered as confounding risk factors among PD population (48, 49). Therefore, in PD patients the preexisting comorbidity such as hypertension, diabetes, and heart failure increases the risk of severe COVID-19 (50).

Another aspect to consider is the impact of SARS-CoV-2 outbreak in the PD management. COVID-19 had a detrimental effect on the in-patient management of PD population such as the difficulty in procuring medication and inability to access in health care. Indeed, as showed early by Bhidayasiri in the early phase of pandemic, PD patients with device-aided therapies (e.g., deep brain stimulation, apomorphine and levodopa-carbidopa intestinal gel infusion) encountered problems because of elective procedures were almost excluded from hospital admissions during lockdown (51). Also, since PD patients are vulnerable, the risk of getting SARS-CoV-2 infection during hospitalization was not negligible during the SARS-CoV-2 outbreak and this also may have limited the hospitalization of these patients since the risk of hospitalization was higher than the benefits. On the other hand, PD should be protected by SARS-CoV-2 infection since hospitalization may lead to detrimental effects such as delirium, adverse drug reactions and aspiration pneumonia in these patients (52). These arguments are true also for other neurological disease. Indeed, it has been showed that patients presenting with ischemic stroke during the COVID-19 pandemic lockdown period had a reduced hospital care and higher hospitalization costs although the final outcome was unchanged compared to the period before lockdown (53). If the access at the emergency department is mandatory in the case of stroke, since PD is a chronic disease, telemedicine including remote delivery of treatment (e.g., ParkinDANCE program) proved to be successful in providing medical services to patients with PD (54, 55).

Although the impact of COVID-19 among the PD population is controverse (Table 1) and it needs to be better assessed in the future and the risk of severe COVID-19 in PD is still not clear, we agree in considering PD patients as vulnerable subjects during COVID-19 outbreak.

**Table 1:** Major findings of the clinical and biological relationship between PD and COVID-19.

Features	Major findings	Reference
Frequency of PD after COVID-19	Unknown. Parkinsonism reached a prevalence of 0.46% within 6 month from COVID-19 recovery.	(56)
Frequency of COVID-19 in patients with PD	No significant difference between patients with PD and the general population	(57–59)
	Higher in patients with PD (0.9% vs. 0.35%)	(60)
	Higher in patients with PD (1.1% vs. 0.6%) especially in patients ≥65 years of age	(61)
	Higher risk of SARS-CoV-2 among PD patients (HR = 1.3)	(62)
Outcome of COVID-19 in patients with PD	Advanced PD stage and elderly are risk factors for severe COVID-19	(63, 64)
	Longer PD duration was associated with higher mortality	(65)
	Similar outcome between patients with PD and the general population	(66)
	Similar hospitalization, ICU admission and mortality rates between PD patients and the general population	(67)
	Increased mortality in inpatients with PD (35% vs. 20%)	(61, 68)
	Higher risk of 30-day mortality in PD patients compared to controls	(69)
	Higher risk of hospitalization in PD patients but similar mortality compared with general population	(62)
	Preexisting comorbidity such as hypertension, diabetes, heart failure increases the risk of severe COVID-19 in patients with PD	(70)
Impact on PD symptoms	Worsening of motor and non-motor symptom or complaining of new motor or non-motor symptoms	(28, 58, 63, 65, 71–73)
	Bradykinesia was the most common worsened symptom followed by gait disturbances, tremor, and rigidity.	(74)
	Worsening of MDR-UPDRS score during pandemic era	(75)
	Worsening of sleeps-disturbances, cognitive function, autonomic function, mood disorders, appetite disorders and pain	(71)
	Fear about own and family, depression due to job difficulties, constant worrying due to COVID-19, low energy, restlessness, clenched jaw, nervous behaviors were common in patients with PD	(76)
Impact of social restrictions	Difficulty in procuring medication and decreased healthcare services and physiotherapy	(77–80)
	Worsening of balance, cognition and IADL	(81)
	Worsening of symptoms during lockdown in over 40% of 2,500 PD patients with especially tremor, pain, and rigidity	(82)
	Higher depression and anxiety in patients with PD and their caregivers due to interruption of non-pharmacological therapy, COVID-19 worry, interruption of outpatient clinic	(83)
	Telemedicine including remote delivery of treatment proved to be successful in providing medical services to PD patients	(84, 85)

PD, Parkinson’s disease; ICU, intensive care unit; IADL, instrumental activities of daily life; MDR-UPDRS, Movement disorder Unified Parkinson’s disease rating scale.

## Shared clinical features between COVID-19 and PD

**Figure 2:**
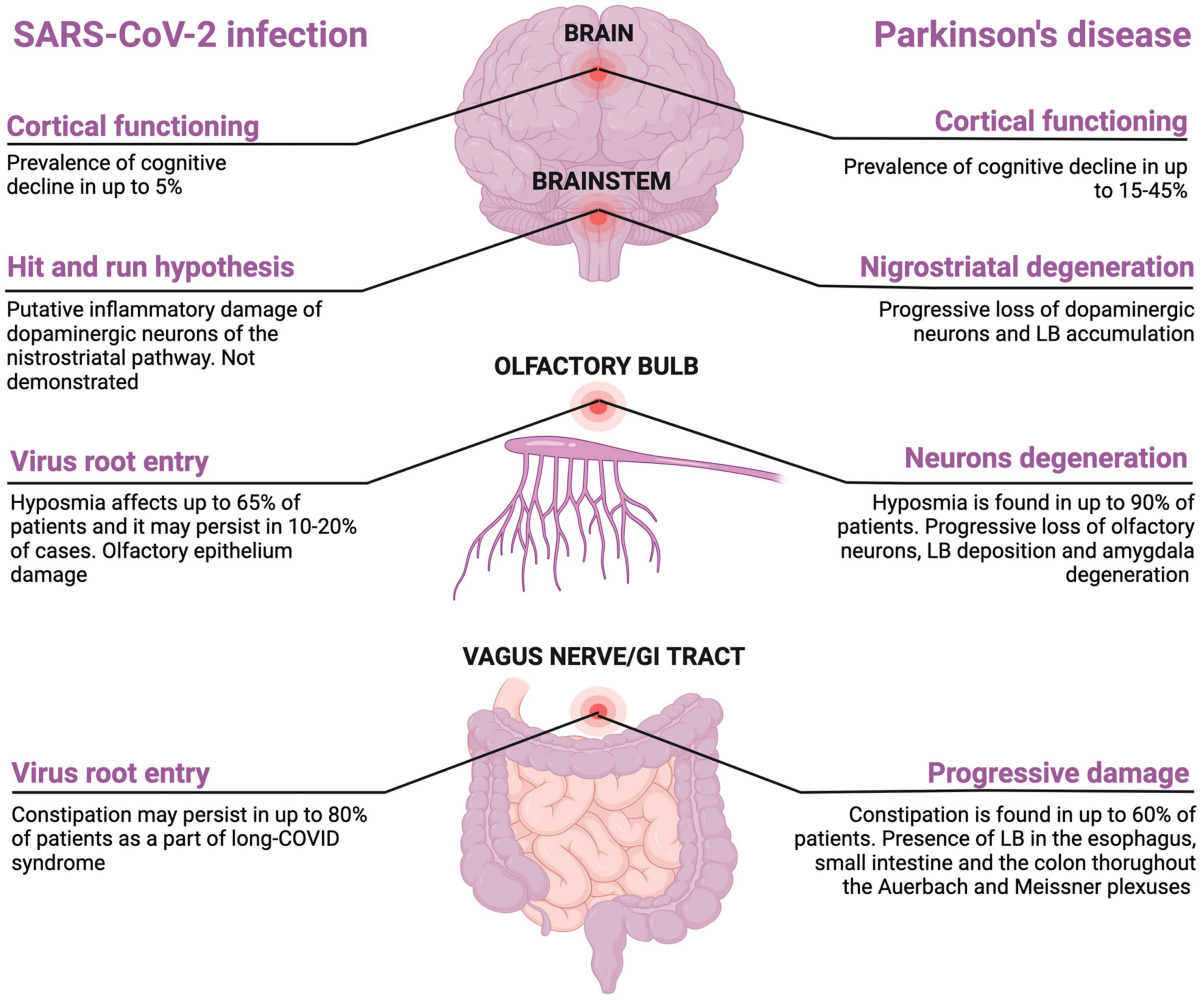
Shared clinical features between SARS-CoV-2 and Parkinson’s disease. Created with BioRender.com.

Currently, many reports exist describing cases of parkinsonism following SARS-CoV-2 infection (37, 86–89). However, these case reports pointed out how parkinsonism may be a consequence of SARS-CoV-2-related encephalitis but did not show a clear link between COVID-19 and idiopathic PD. As the relationship between acute or secondary parkinsonism and SARS-CoV-2 infection is beyond the purpose of this review, we will focus our attention only on the association between SAR-CoV-2 and idiopathic PD. Although the incidence and the prevalence of PD in people who experienced COVID-19 are not clear and the association between SARS-CoV-2 infection and PD is purely putative, there are several common clinical features between PD and COVID-19 (Figure 2). It has been well-established that COVID-19 in PD patients may worsen both motor symptoms such as bradykinesia, rigidity, balance disturbance as well as non-motor symptoms (e.g., motivation, intellectual impairment) (45). Of note, bradykinesia was the most common worsened symptoms followed by gait disturbances, tremor, and rigidity (90). Overall, a worsening of Movement Disorder Society Unified Parkinson’s Disease Rating Scale (MDS-UPDRS) was noted in pandemic era in PD patients (91).

A study including 5,429 PD patients revealed the worsening of non-motor symptoms such as sleeps-disturbances, cognitive function, autonomic function, mood disorders, appetite disorders and pain (92). Also, emotional symptoms of stress due to COVID-19 concerns are not negligible in PD patients (93).

Around the 65% of people with COVID-19 experience hyposmia that is also a common prodromal symptom of PD affecting up to 4% of patients (94, 95). Olfactory dysfunction after COVID-19 recovery may persist in 10 to 20% of patients which may indicate a permanent loss of the renewing olfactory neurons, phenomenon that is also seen in PD patients (96). Given the increased incidence of encephalitis lethargica following Spanish flu in 1920s, it has been theorized that neurodegenerative PD may represent a clinical manifestation of long COVID (97). This hypothesis may be supported by the increasing evidences that COVID-19 may precipitate a neurodegenerative process leading to a parkinsonism (98–100). In addition, the finding of Lewy bodies in the brain of rhesus macaques infected with SARS-CoV-2 may support the link between neurodegenerative PD and COVID-19, although SARS-CoV-2 RNA has never been found in these macaques (101). This finding may be in line with the “hit-and-run” hypothesis according to which a neurotropic virus triggers an autoimmune reaction leading to chronic inflammation and brain damage (first hit), making it more vulnerable to further neurodegenerative changes (second hit) (102, 103). The degeneration located at the nigrostriatal level may be explained by the idea that dopamine neurons are highly susceptible to systemic inflammation thus explaining the clinical phenotype of this post-infective sequelae (104). To date, the “hit-and-run” hypothesis has been proposed for Spanish Flu in 1920s, H1N1, H5N1, EBV, and HSV-1 (105). Another link between PD and SARS-CoV-2 infection is the gastrointestinal tract involvement as constipation may precede motor symptom in PD, symptom that also persists in up to 80% patients after COVID-19 as long COVID symptom (106, 107). Cognitive decline affects 15-40% of patients with PD (108). There are growing evidences suggesting cognitive decline as long-term consequence of COVID-19 infection. Taquet et al., showed a dementia prevalence of 5% among COVID-19 survivors aged more than 65 years within 6 months after SARS-CoV-2 infection which was higher compared to one after influenza and other respiratory tract infection (109). A cross-sectional online study including 85.000 participants revealed that the degree of cognitive decline in patients with a history of severe COVID-19 was equivalent to the loss of 10 years compared to healthy controls (110, 111). However, the association between COVID-19 and cognitive decline require longitudinal studies with long-term follow-up. Indeed, the observation period from COVID-19 outbreak and the potential detrimental effect on cognitive function is limited and it did not allow to estimate with certainty the risk of cognitive decline in patients who experienced COVID-19. Also, participants included in that studies were older, and the age-dependent risk of cognitive decline cannot be excluded. In Figure 2 the common features between SARS-CoV-2 and PD are reported. There are other points to discuss: (1) it is questionable to ask what the prevalence of COVID-19 in people with PD is and what is the magnitude of the infection in these patients; (2) it is interesting to know how PD affects the management of COVID-19 and how COVID-19 affects the therapeutic management of PD; (3) finally, the impact of the lockdown on motor and non-motor symptoms in these patients should not be neglected.

## From neurological complications of SARS-CoV-2 to its neurotropism

The recent infection by SARS-CoV-2 is clinically characterized by fever, cough, and shortness of breath, associated with the development of an acute respiratory distress syndrome (49). However, several reports highlighted neurological complications following SARS-CoV-2 infection involving both central (CNS) as well as peripheral nervous system (PNS). First descriptions of neurological manifestations occurring in patients with COVID-19 have been described in a series of patients in Wuhan, reporting a prevalence of neurological symptoms in up to 45% of patients with more severe disease (112). Neurological symptoms more commonly were vague and consisting in headache, dizziness, and myalgia but they were possibly more severe involving both central nervous system (CNS) (impaired consciousness, acute cerebrovascular disease, ataxia, and seizures) (113) and peripheral nervous symptom (PNS) (taste, smell, and vision impairment, and neuralgia) (114). Of the 125 patients reported in the first surveillance study of acute neurological and psychiatric complications of COVID-19, 77 (62%) presented with a cerebrovascular accident (most commonly ischemic stroke), 39 patients with altered mental status (31%) (9 patients with unspecified encephalopathy, 7 patients with encephalitis). Interestingly, of the 39 patients with altered mental status, most of them (59%) fulfilled the clinical case definition for psychiatric diagnoses (10 new onset psychosis, six neurocognitive dementia, 4 affective disorder). Of the remaining 9 patients, 6 had a peripheral disorder and 3 other neurological symptoms (respectively opsoclonus-myoclonus syndrome, sixth nerve palsy, and seizures) (115). It remains to be clarified whether the damage caused by the virus to the nervous system is direct connected to the invasion of neurons by virions, or rather it is indirect such as post-infectious mechanisms (116). In this case, the mechanisms of molecular mimicry, epitope spreading, and bystander activation may clarify how SARS-CoV-2 infection triggers an autoimmune reaction throughout the CNS (i.e., viral-induced autoimmunity) leading to autoimmune encephalitis with the finding of antibodies against onconeural and surface antigens (e.g., N-Methyl-D-Aspartate Receptor, voltage-gated potassium channel, amphiphysin, myelin oligodendrocyte glycoprotein, etc) as highlighted recently by Stoian et al. (65). The autoimmune hypothesis could also be suggested by the improvement after immunosuppressive therapy in some patients with movement disorders following Sars-Cov-2 infection (11).

The hypothesis of neuroinvasion by Sars-Cov-2 comes from the finding of SARS-CoV-2 RNA in the CSF of patients with COVID-19 (66). Possible routes of invasion of the virus into the CNS, are the olfactory epithelium (OE), the olfactory bulb (OB) and the brain endothelial cells (ECs). However, it has been shown as the finding of viral genome or intrathecal antibody synthesis in patients affected by COVID-19 who experienced acute neurological symptom is rare (56). Moreover, studies from autopsies conducted on the brains of patients who died from COVID-19 have rarely shown a presence of the virus in the cerebral parenchyma (117) and some authors did not find presence of SARS-CoV-2 both at genomic and antigenic levels in the brain (118). It is possible to hypothesize a mechanism of damage on the CNS similar to that of the 2009 H1N1 pandemic (CA/09) influenza virus which, while not infecting neurons, is capable of inducing the death of nerve cells, also including the dopaminergic neurons of the substantia nigra, through the induction of a potent inflammatory response (57). However, some authors have found the presence of the virus directly in the CNS. Indeed, in a group of 24 COVID-19 patients who died of respiratory failure, the virus was detected in the dorsal cord and substantia nigra of five COVID-19 subjects but not in controls, but the neurodegenerative potential of this finding needs further investigation (60). Therefore, neuroinflammation and protein interactions are the two mechanisms considered most plausible to explain a possible neurodegenerative effect on dopaminergic neurons caused by SARS-CoV-2.

## The olfactory entry route in the nervous system of SARS-CoV-2

The mechanisms underlying SARS-CoV-2 neuro-invasion are not yet fully understood. It has been shown that SARS-CoV-2 is able to infect and replicate in cultures of human neural progenitor cells and human induced pluripotent stem cells (hiPSCs)-derived brain organoids via ACE2 (69). The expression of this receptor in comparison with other tissues is not very high in the CNS (67). In the CNS, ACE2 is distributed mainly in the spinal cord, in the substantia nigra, in the hippocampus and in the hypothalamus (63). The interaction between SARS-CoV-2 and ACE2 is facilitated through the priming of protein S by transmembrane serine protease 2 (TMPRSS2) (64). However, ACE2 and TMPRSS2 do not frequently co-localize in the brain. Differently, the sustentacular non-neuronal cells of the olfactory epithelium (OE) have a high level of co-expression of ACE2 and TMPRSSS2 (62). It is therefore possible that OE represents a point of penetration with a subsequent diffusion of Sars-CoV-2 within the brain, although the ways of propagation of the virus are not yet known. In models of ACE2 Knock-in mice, the localization of SARS-CoV-2 at the level of the olfactory bulb (OB) is already observable in the fourth post-infection day. This site is probably reached by retrograde axonal transport starting from the OE of nasal cavity. It is important to underline that this type of neuro-invasion can also occur regardless of passing through the blood brain barrier (BBB). In fact, even in the absence of viraemia SARS-CoV-2 is able to invade the OB (119). It is therefore not surprising that a link to neurological disorders turns out from the reduction or the loss of sense of smell (hyposmia or anosmia), symptoms of COVID-19 pandemic, fully recognized among those belonging to the SARS-CoV-2 infection (70). The hyposmia observed during SARS-CoV-2 infection might have various origins. It can result from direct damage to the sustentacular cells which are the cells that express the highest concentration of ACE2 at the OE level or from indirect damage to nearby olfactory receptor neurons due to the release of inflammatory cytokines and of the immune response induced by the virus (120). Autopsy studies have shown a poor localization of SARS-CoV-2 in the olfactory receptor neurons of the OE cells that do not normally express high ACE2 levels (74) (62). However, mRNA localization of SARS-CoV-2 in neurons should be investigated in the future (75). Moreover, the uptake of spike subunit 1 (i.e., S1) in OB is greater after intravenous administration than intranasal administration probably because invasion of the brain by SARS-CoV-2 occurs independently of the ACE2 receptor (71). It is therefore possible that the loss of the olfactory receptor neurons occurs after invasion of the OB due to anterograde degeneration of the axon (76).

## Blood brain barrier allows SARS-CoV-2 penetration into the brain

Several evidences suggest that the S1 protein allows the SARS-CoV-2 to enter the brain through the bloodstream. ACE2 has an important role in allowing the uptake of the virus thanks to the wide range expression of the receptor into the brain endothelial cells (ECs) (63). In particular, the arginine-glycine-aspartate motif of the S1 protein interacts with the α5β1 integrin expressed on brain ECs. Subsequent activation of the mitogen-activated protein kinase pathway could facilitate virus entry into the endothelial cells (ECs) (81). Rhea and collaborators had shown that in normal mice S1 subunit of the spike protein is able to cross the BBB thanks to the transcytosis mechanisms and to localize in various areas of the brain such as basal ganglia or many regions of the cerebral cortex (71). Moreover, several *in vitro* models of human BBB highlighted that S1 can penetrate the endothelial barrier (77). The group of Krasemann showed that crossing of the BBB by SARS-Cov-2 seemed not to occur by compromising the paracellular tight junctions of brain capillary endothelial cells but this happened through mechanisms of transcellular transport across the cells from the apical side to the basolateral compartment (78). Similar result was obtained by other researchers (77). However, Yang also revealed a remodeling of the tight junctions and a consequent increased permeability of BBB after SARS-Cov-2 infection of the brain microvascular endothelial cells (83).

## Neuroinflammation induced by SARS-CoV-2: a link to neurodegeneration?

Brundin and colleagues hypothesized that the neuroinflammation caused by SARS-Cov-2 may result in the loss of dopaminergic neurons (99) Chen showed that SARS-CoV-2 can infect and induce inflammation and senescence of dopaminergic neurons derived from hiPSCs (82). The persistence of neuroinflammation after infection can influence the development of neurological sequelae. Analysis of the content of neuronal extracellular vesicles isolated from plasma of 24 patients recovering from COVID-19 showed the presence of marker proteins of neuronal dysfunction such as amyloid beta, neurofilament light, neurogranin, total tau, and p-T181-tau in greater quantities compared to healthy controls (84). In a cell culture model, it was showed that spike protein can modify the content of exosomes released by SARS-CoV-2 transfected cells. In fact, infected cells release a great number of exosomes enriched of some microRNAs such as miR-148 causing neuroinflammation thanks to the activation of the human microglia (85). Indeed, microglial cells express the ACE-2 receptor (121) and these cells are hyper-activated by SARS-CoV-2, although the presence of the virus has not yet been demonstrated *in vivo* inside them (122). Biopsy analysis of patients who died from COVID-19 showed widespread activation of microglia in several areas of the brain, especially in brainstem, while proinflammatory microglia in the hippocampus were remarkable only if the course of COVID-19 was complicated by delirium (123). It might be hypothesized that a possible link between development of PD and SARS-CoV-2 infection may involve microglia. In fact, it is known that the pro-inflammatory microglia play an important role in the loss of dopaminergic neurons. Another pathogenetic link between PD and COVID-19 is related to similar inflammatory pathways that the two diseases share (124). Some researchers suggested that NF-κB-associated inflammatory pathways are induced by SARS-CoV-2 infection and it can also lead to death of the dopaminergic neurons (124). However, more studies are needed to analyze the pathways of this interaction in more detail. A further possible link between PD-related neuroinflammation and COVID-19 could arise from the fact that COVID-19 and Gaucher disease (GD) share upregulation of complement 5a (C5a) and its C5aR1 receptor and excessive glycosphingolipids synthesis with subsequent activation of the immune system and generation of pro- inflammatory cytokines. GB is caused by mutations of the GBA gene, which is also a risk factor of PD, so it is possible to hypothesize that a similar mechanism is also shared by PD with GBA mutation and COVID-19, not only by GD and COVID-19 (125).

Dysfunction of mRNA metabolism, such as its transport or degradation, may play a role in neurodegenerative mechanisms (126). Some authors have hypothesized a possible neurodegenerative mechanism linked to the spike protein which would acquire prion-like properties. In particular, according to these authors, following vaccination with mRNA vaccines for Sars-CoV-2, the spike protein could be produced in excessive quantities by cells and interact with other prion-like proteins leading to the formation of aggregates (127). It could be hypothesized that this mechanism also occurs following viral infection by Sars-Cov-2 and could be connected to neurodegeneration.

## SARS-CoV-2-proteins interaction: evidence and hypothesis

It has been hypothesized that the vulnerability to PD development conferred by SARS-CoV-2 might derive from the ability of viral proteins to alter some proteins expressed in the lung and disrupt protein–protein interaction in the CNS. Proteins released from the lungs, due to the greater pulmonary permeability during inflammation induced by SARS-CoV-2 infection, would be transported in the circulation by exosomes to the brain, where, thanks to their alteration produced by viral components, could perturb protein with a role in the development of PD (128). The main protein involved in these mechanisms currently appears to be α-synuclein. The expression of α-synuclein is increased in neurons following a viral infection and its aggregation induced by RNA virus could serve to trap the virus and limit viral replication (129). Conversely, one could hypothesize that this mechanism can increase the risk of developing PD. It is important to note that α-synuclein is expressed not only in neurons of the CNS but also in those of the PNS, including neurons of the enteric nervous system (130). It has been hypothesized that α-synuclein may serve to inhibit the neuro-invasion of viruses from the PNS to the CNS (131). SARS-CoV-2, a RNA virus, is able to infect the neurons of the myenteric plexus due to the high expression of ACE2 in these cells (132). The overexpression of α-synuclein in SNP cells has been hypothesized to constitute a defensive mechanism that seeks to circumscribe the virus but also an alteration that predisposes to the subsequent development of PD according to the Braak hypothesis of the disease (133). Recently, in order to find a possible molecular link between SARS-CoV-2 infection and the development of PD, the group of Semerdzhiev showed that the spike protein fails to induce the aggregation of α-synuclein, while protein N has the ability to induce precipitation of α-synuclein into the amyloid fibrils characteristic of PD (134). The immunohistochemical analysis of the skin biopsy samples from five patients who experienced postural tachycardia syndrome after SARS-CoV-2 infection showed the presence of the α-synuclein in a phosphorylated form, the pathological state of α-synuclein in PD or dementia with Lewy bodies, multiple system atrophy and pure autonomic failure (135). However, COVID-19 patients experiencing neurological symptoms possess serum and CSF alpha-synuclein levels comparable to those of COVID-19 patients without neurological manifestations (136). Therefore, more data are needed to clarify the role of α-synuclein induced by viral infections such as SARS-CoV-2 as a risk factor for PD symptoms development.

## Anti-parkinsonian drugs in the pandemic era

A brief discussion should be reserved for some anti-parkinsonian drugs during the pandemic era. It has been reported that dopamine agonists may be protective against severe COVID-19 although another study did not show this benefit (45, 137). However, the mechanism underlying the benefit effect of dopamine agonist against severe COVID-19 is unknown. It would seem that dopamine agonists are capable of inducing an inhibition of Sars-CoV-2 replication, mainly through an action on the D2 receptor, recapitulating the antiviral effects of dopamine (Limanaqi F et al., 2022). Moreover, it must be considered that generally dopamine agonists are used in the initial or mild stages of the disease, therefore in patients less advanced in the natural history of PD. Also, amantadine, an anti-viral drug used to treat levodopa-induced dyskinesia has been hypothesizing to disrupt the lysosomal machinery needed for SARS-CoV-2 replication or to block the viroporine channel of SARS-CoV-2 preventing the release of the viral nucleus into cells (58, 59, 61). Accordingly, to these hypotheses, several studies has been published indicating the potential role of amantadine in the prevention of the clinical symptoms related to COVID-19 (68, 72). Some authors showed that patients with PD taking amantadine had lower COVID-19-related mortality (72) or a lower rate of SARS-CoV-2 infection (73) compared to those who were not taking it. Although it is not an antiparkinsonian drugs, a brief consideration should be reserved to the mysterious relationship between vitamin D, PD and COVID-19. Patients with PD often have reduced levels of vitamin D (79). The group of Fasano and colleagues also observed a higher percentage of unaffected PD patients in the vitamin D group than in the control group who did not take vitamin D supplementation (41). Daily intake of 2,000-5,000 IU of vitamin D3 is able to slow the clinical progression of disease in patients with PD and also offers the advantage of protecting patients from COVID-19 (80). These observations agree with the fact that vitamin D has been shown to possess some antiviral properties (138) and neuroprotective effects. In fact, studies on rodents have shown that the supplement of vitamin D not exceeding the toxicity levels is able to reduce the extent of the loss of dopaminergic fibers induced by toxic substances (139). Moreover, vitamin D is able to reduce inflammation by decreasing the production of proinflammatory cytokines such as interferon-gamma and tumor necrosis factor-alpha (140, 141). This likely could be a common point in both PD and COVID-19, since the inflammation associated with cytokine hyper-production often coincides with clinical worsening during the course of COVID-19 (142, 143) and at the base of PD there are also important inflammatory mechanisms (144). However, even in this case the results are conflicting because there are several studies that have not shown any ability of vitamin D to influence the course and outcome of COVID-19 (145).

## Conclusion

The link between SARS-CoV-2 and PD is multi facet. Whether the higher prevalence and incidence of PD in patients who experienced COVID-19 are controverse, the hypothesis that COVID-19 may increase the risk of PD is actually unlikely. On the other hand, patients with PD showed a worsening of motor and non-motor symptoms during COVID-19 outbreak due to both infection and/or social restriction. Also, due to the nature of neurodegenerative disease, older age and comorbidities, PD patients are vulnerable during the COVID-19 outbreak since they reported higher mortality and morbidity compared to healthy population. Many are the clinical and pathogenetic shared features between SARS-CoV-2 infection and PD although much remains to be clarified about this complex and bidirectional relationship. Although cases of acute parkinsonism following SARS-CoV-2 infection are reported, the possibility of higher risk of occurrence of idiopathic PD following SARS-CoV-2 infection is still controverse. Current data suggesting the development of idiopathic PD associated with SARS-CoV-2infection is currently only a hypothesis. Of course, due to the short interval period from SARS-CoV-2 outbreak we cannot have reliable data about the incidence of idiopathic PD among infected people and therefore we cannot estimate the risk to develop PD after COVID-19. Longer longitudinal studies are needed to better clarify the relationship between COVID-19 and the development of idiopathic PD. Looking at the several shared featured between SARS-CoV-2 infection and idiopathic PD pathogenesis, we believe that prospective evaluation of patients with permanent hyposmia and gastrointestinal symptoms after COVID-19 might help to better clarify the relationship between COVID-19 and idiopathic PD incidence.

## Author contributions

MD’A, SI, and GS contributed to the conception and design of the study. SI and GS wrote the first draft of the manuscript. MD’A contributed to the validation, revision, editing, and supervision. MD’A, SI, GS, CD, SM, MA, FG, VA, PA, PR, CG, CC, and NS performed the search and validation of literature data. All authors contributed to the manuscript revision, read, and approved the submitted version.

## Conflict of interest

The authors declare that the research was conducted in the absence of any commercial or financial relationships that could be construed as a potential conflict of interest.

## Publisher’s note

All claims expressed in this article are solely those of the authors and do not necessarily represent those of their affiliated organizations, or those of the publisher, the editors and the reviewers. Any product that may be evaluated in this article, or claim that may be made by its manufacturer, is not guaranteed or endorsed by the publisher.
